# Supplementary material for: Learning a deep language model for microbiomes: The power of large scale unlabeled microbiome data
Source: PLoS Comput Biol. 2025 May 7;21(5):e1011353. doi: 10.1371/journal.pcbi.1011353 (PMC12058177; doi:10.1371/journal.pcbi.1011353)
Supplement: Attachment — Submitted filename: Microbiome DL Paper Reviewer Feedback.pdf [file pcbi.1011353.s004.pdf]

Dear editor,

We again thank the reviewers for their further feedback. Our itemized response follows, with reviewers' original comments italicized and our responses shown in blue below each comment.

Reviewer #1:

The authors have addressed all the comments. It is suggested to accept this manuscript for publication

We thank the reviewer for their time and careful evaluation of our manuscript. We are pleased that our revisions have successfully addressed all previous concerns and appreciate their recommendation for publication.

Reviewer #4:

# Major comments

While Pope, Varma et al., have definitely improved the manuscript, I find the manuscript still lacking in a few areas, which are listed below. I believe that these items should be addressed prior to publishing the article.

## Dataset split

I thank the authors for clarifying. I should note that randomly spliting the dataset, instead of using cross-fold validation can easily result in less reliable performance metrics. However, the dataset is somewhat large, so this potential issue is less of a concern.

We initially attempted K-fold cross validation but moved to independent data splits due to statistical concerns. With multiple samples per patient that must remain together, achieving balanced class distributions across all K-fold combinations proved impossible through patient-level random assignment. Forcing such balance would require highly specific, non-random patient assignments to folds. This artificial constraint would introduce unknown correlations between different cross-validation experiments, potentially biasing our performance estimates and compromising the statistical independence assumptions underlying K-fold CV. Independent splits, while not leveraging all data simultaneously like K-fold CV, maintain proper randomization and independence between experiments, providing more reliable performance estimates.

## Dataset Skew

Given that AUC focus on TPR vs. FPR, 90% true positives can be misleading, since the FPR will be calculated from a small number of negative instance (DOI:10.1145/1143844.1143874).

The authors state: "… we do not want to have to re-adapt our model to new class skews during

testing on novel datasets.”; however, real-world datasets will likely substantially differ in label skews (e.g., datasets with a low number of true positives, unlike the Halverson and HMP2 datasets). The authors have not shown whether their model can generalize to datasets with low prevalences of true positives. This detracts substantially from the impact of the work.

We appreciate the reviewer's concern about model performance on datasets with different class prevalence. However, we want to clarify that our primary contribution is not proposing a diagnostic tool, but rather establishing a framework for learning meaningful microbiome embeddings that can be adapted to various downstream tasks. The IBD prediction task serves as one concrete test case demonstrating the utility of our learned representations.

That said, we did evaluate our approach's robustness across datasets with different characteristics: The AGP training data has ~5% IBD prevalence, while HMP2 and Halfvarson have ~79% and ~90% IBD prevalence respectively. Our model's strong performance across these varying scenarios suggests the learned representations capture genuinely useful biological signals rather than just dataset-specific patterns.

The reviewer raises a valid point that real-world datasets may have even lower disease prevalence. While exploring extremely low prevalence scenarios would be valuable future work, it's somewhat tangential to our core contribution of demonstrating that transformer-based language models can learn biologically meaningful microbiome representations, as evidenced by our robust prediction results, our analyses of the learned embeddings' structure, and the correlations with known metabolic pathways.

Our focus was on establishing proof-of-concept for this novel methodology, which we believe the current results accomplish. The framework we've developed could be adapted and optimized for specific downstream research scenarios with different prevalence requirements as needed.

More generally, the authors did not directly compare their model to standard microbiome machine learning baselines. The other models tested were dimension reduction or deep learning approaches, but there was no comparison to statistical machine learning models (e.g., linear, random forest, or SVM), trained directly on microbiome features (e.g., ASVs or ASVs aggregated at the genus or family taxonomic level). Literature such as https://www.biorxiv.org/content/10.1101/2024.09.16.613342v2, https://doi.org/10.1038/s41467-022-34405-3, and https://doi.org/10.3389/fmicb.2023.1261889 has shown that simple models can often outperform substantially more complicated deep learning approaches, in the domain of biology when dataset size is limited. Given this literature, and the lack luster performance of the authors’ model versus the other models assessed (Table 4), I am skeptical on whether the authors’ model would out-perform a simple random forest model trained on taxon abundances (or presence-absence).

We appreciate the reviewer's suggestion about comparing to standard ML baselines, and have incorporated the suggested baseline in our experiments. As expected, the results show that Random Forests trained on 26,726-dimensional abundance profiles achieve strong in-domain

performance. However, they suffer significant performance degradation when tested on different study populations, with the decline becoming more pronounced as the divergence between the training and testing populations increases. In contrast, our model remains robust across populations, maintaining strong performance despite shifts in microbial composition, highlighting its ability to capture fundamental microbiome patterns that generalize beyond dataset-specific correlations.

Importantly, we would like to clarify that the primary goal of our work is not to develop the most accurate IBD prediction models. Instead, our main contribution is introducing a method for learning meaningful, generalizable representations for microbiome data that capture biological structure and can adapt across populations and tasks. Our reported performance results were always intended to serve as evidence for having achieved this goal, rather than being the goal.

Our prior writing was unclear on this point. We have revised the abstract, introduction and author summary to better emphasize the paper's objective and show how multiple converging lines of evidence validate that our learned representations preserve relevant biological signals. To summarize:

1. **Phenotypic relevance:** Supervised learning results on IBD and dietary tasks confirm the embeddings preserve phenotype-relevant information

2. **Cross-population generalization:** Strong cross-population generalization shows the representations capture fundamental microbiome patterns rather than dataset-specific correlations

3. **Taxonomic structure:** Analysis of the embedding space reveals it naturally captures taxonomic relationships, despite never being trained on phylogenetic information

4. **Attribution analysis:** Attribution analysis identifies biologically meaningful taxa driving IBD predictions

5. **Correlation with known metabolic pathways:** Correlation analysis shows embedding dimensions align with known metabolic pathways without any explicit pathway training

The authors could argue that their approach is more interpretable than random forest or other statistical ML models; however, I find the authors' demonstration of interpretabliy to lack rigor. The description of Fig 5 includes an abundance of "seems to be" and "appear to" phrases. More generally, the interpretive ability of t-SNE and UMAP have been called into question (DOI:10.1038/s41467-019-13056-x, DOI:10.1038/nbt.4314); thus, it is unclear whether the following descriptions in the manuscript of the t-SNE ordinations are useful: "appear to lie on long 'strands'" and "made up of smaller 'threads' very close together".

We appreciate the reviewer's concern about the rigor of our interpretability analysis. We employ t-SNE to intuitively visualize the **local** structures captured by our embeddings, and its alignment with taxonomic organization. Since t-SNE is designed to preserve local neighborhoods while

potentially distorting global relationships (Van der Maaten & Hinton, 2008, Kobak & Berens, 2018), we believe it is appropriate for this purpose.

However, we acknowledge the potential limitations of t-SNE, particularly the risk of visualization artifacts. To address this, we have provided additional quantitative analyses of the taxonomic structure in the original embedding spaces. Specifically, Figure 4 (c) presents K-means clustering results across different K values, showing that the average phylum purity of clusters in our embeddings is approximately 20 percentage points higher than in GloVe embeddings. This confirms that the observed phylogenetic clustering is not merely a visualization artifact, but rather a meaningful property of our embeddings, which exhibit stronger alignment with taxonomy. In our revision, we have provided a more detailed explanation of this analysis and its findings.

Kobak, D., & Berens, P. (2018). The art of using t-SNE for single-cell transcriptomics. *Nature Communications, 10*.

Maaten, L.V., & Hinton, G.E. (2008). Visualizing Data using t-SNE. *Journal of Machine Learning Research, 9*, 2579-2605.

I previously asked for more quantitative approaches to interpreting the results (e.g., clustering or comparing taxonomic distance to embedding distance), but the authors have not included such analyses.

See response above.

## Methods

I thank the authors for clarifying the methods. I stil do not find the heatmaps in Fig 6 to be useful; it is quite hard to discern a qualitative difference. The same applies to Fig 7, in that the distributions are quite similar. While the K-S and E-S tests show low P-values, what is the effect size? Moreover, I believe both tests assume independent data points, which seems to be violated for the embedding data.

To address the reviewer's concerns about independence assumptions and effect size, we ran a permutation test, which makes minimal assumptions about the data's structure.

For each permutation, we:

1. Pooled all correlation values
2. Randomly split into groups matching original sizes
3. Calculated difference in mean correlation magnitude

After ten million permutations, we found that not a single permuted sample produced a difference of means as large as our observed difference of 0.0185, indicating $p < 10^{-7}$. The effect size, as measured by Cliff’s delta, is 0.2.

Regarding the heatmaps in Fig 6, we agree these are primarily useful for visualizing the distribution of correlations rather than demonstrating quantitative differences. We are happy to move it to the Supporting Information section if the reviewer finds them distracting from the main message.

## Code

While the authors state that they have provided their code, I could not find it.
The authors should provide all of their code, and the code should be well-organized so that the work can be fully replicated.

The code is available at the Dryad repository we previously provided to the reviewer, under the "Software" tab, which links to https://doi.org/10.5281/zenodo.13858903. We apologize for any confusion and will ensure the README clearly points to this location.

# Minor comments

Line 101: "a bacteria" => "a bacterium"
Line 518: "By comparing the observed correlations to this null distribution, we filtered out correlations that were not statistically significant" => what is "statistically significant" in this context?

We have also corrected two minor issues:

1. Line 101: Changed "a bacteria" to "a bacterium"
2. Line 518: Clarified statistical significance as correlations with $p < 0.001$ against the simulated null distribution

The significance testing for correlations between embedding dimensions and pathways uses a permutation-based approach with 1,000 permutations. For each embedding dimension, correlations with pathways are considered significant only if they exceed all values in the null distribution generated through permutation (effectively $p < 0.001$).
