# Supplementary material for: Learning a deep language model for microbiomes: The power of large scale unlabeled microbiome data
Source: PLoS Comput Biol. 2025 May 7;21(5):e1011353. doi: 10.1371/journal.pcbi.1011353 (PMC12058177; doi:10.1371/journal.pcbi.1011353)
Supplement: Attachment — Submitted filename: Second Microbiome DL Paper Reviewer Feedback.pdf [file pcbi.1011353.s005.pdf]

Dear editor,

We again thank the reviewers for their additional feedback. Our itemized response follows, with reviewers' original comments italicized and our responses shown in blue.

Reviewer #1:

The authors have addressed most of the reviewer's comments. Here are a few minor points for this manuscript.
1. Please highlight the best performance in Table 3. For those baselines proposed by other research works, please add the corresponding citations.

We have highlighted the best performing methods in tables 3 and 4. We have also added relevant citations for the methods introduced by DeepMicro, as well as our other baselines.

2. The authors perform data augmentation by randomly deleting 10% of the input taxa in each training sample to reduce overfitting. Could the authors give citations to this technique or explain more details?

We have added additional details on the dropout method, as well as a relevant citation.

Reviewer #3:

The authors have addressed most of my concerns and the manuscript has been significantly improved.

We are grateful for the reviewer's prior feedback and are glad to see that our changes were able to address their concerns.

Reviewer #4:

# Major Comments

The manuscript presents a novel application of transformer models to microbiome data, demonstrating the potential of leveraging large-scale, unlabeled microbiome datasets to develop robust predictive models. The authors state that their approach shows improved performance in multiple prediction tasks, such as predicting Irritable Bowel Disease (IBD) and dietary patterns, which highlights the effectiveness of contextualized taxa representations. Additionally, the authors provide model interpretations in order to derive biological insight from their methodology.

While I find this work to be novel and potentially impactful, I have many major and minor concerns that I believe should be addressed in order make the manuscript suitable for publication in Plos Computational Biology.

## Dataset Split

It appears that the authors applied GloVe on the entire American Gut Project (AGP) dataset and subsequently used a subset of this AGP dataset for testing (see minor comments below). If true, this approach appears to constitute data leakage, given that GloVe is assessing taxon co-occurrance across all samples, even those in the AGP used for model testing. Such data leakage would thus likely produce an overly optimistic estimation of model performance and generalization.

It is correct that the entire AGP dataset was used for all unsupervised and self-supervised portions of all methods, which includes the GloVe training in Tataru and David, the ELECTRA model pretraining stage in this current work, the PCA dimension reduction baseline and pretraining of the autoencoder baselines. However, we ensured a fair evaluation of our method by allowing the baselines exactly the same access to AGP unsupervised data (with the exception of the Gaussian random projection baseline, which is unable to take advantage of any unsupervised data). We have clarified this point in the text.

We additionally emphasize that exposure to the unlabeled AGP data points does not constitute a leak of the *labels*.

Moreover, I believe that at least for the Halverson dataset, there are multiple samples per individual, so the samples are not independent. It's not clear to me whether part of the Halverson dataset was used for fine-tuning, while the other part was used for valiation (clarification in this regard in the Methods would be helpful). If this is indeed the case, did the authors split the dataset so that all samples belonging to the same individual were solely in the train (fine tune) or test split?

Both Halfvarson and the HMP2 datasets were used purely for testing. We have updated the manuscript to clarify this point. As such, there was no need to split either dataset according to individual.

The same goes for the other datasets; for instance, the AGP definitely includes multiple samples per individual, but it is not clear if the authors blocked by individual when creating the train/test splits.

Each of AGP, HMP2 and Halfvarson include multiple samples per individual. During prior our cross-validation splits, we did not block out training data by individual. We have since switched to blocking out all train / validation / testing splits by individual. As a consequence, we no longer perform 5-fold cross validation because we found it is very difficult to find cross validation fold splits such that all subsequent train / validation / testing splits will have the target class balances. Instead, we perform 5 independent training / validation / testing runs.

We additionally adopted the DeepMicro approach to deciding our stopping epoch for AGP finetuning runs: we find the optimal stopping epoch by using a training / validation split of the non-test data, before converting the non-test data into a combined training set and re-finetune

our model on that data using the previously found stopping epoch.

## Dataset Skew

The Halverson and HMP2 datasets used in this study are highly skewed towards positive classifications for IBD, with 90% and 79% of observations being IBD positive, respectively. This imbalance caps the error rates resulting from false positive classifications and potentially skews the performance metrics. The authors should consider addressing this imbalance, perhaps through techniques such as oversampling the minority class, to ensure that the model performance is not artificially inflated by the dataset skew.

We thank the reviewer for raising this point. In our prior manuscript, we forgot to mention that we balance the training batches by class during finetuning, thus effectively oversampling the minority class. We have clarified this in the manuscript.

However, we do not want to have to re-adapt our model to new class skews during testing on novel datasets. Hence we chose to evaluate our model on the Halfvarson and HMP2 datasets using AUC / AUPR, as they are threshold-insensitive evaluation metrics. We additionally note that dataset skew does not systematically bias the AUC metric (Bradley).

## Methods

The methods section lacks crucial details about the “metabolic pathways” analysis presented in the results. For instance, the paper does not specify how correlations were calculated or the number of ASVs included in the analysis. Additionally, the criteria used for the permutation test to filter correlations are not clearly defined. Providing these details is necessary for understanding the robustness and validity of the findings presented.

We have significantly rewritten our description of the metabolic pathways correlation analysis to include the numbers of ASVs (11,893), the number of pathways (141), and the specific correlation measure used (Spearman).

We compute the Spearman’s correlation between each of our contextualized embedding dimensions and the 141 extracted metabolic pathways, producing a 200 by 141 correlation matrix. The same process is repeated for the 100-dimensional GloVe embedding, producing a 100 by 141 correlation matrix. Figure 6 shows both sets of correlations using heatmaps. We can see that, although both embeddings show clear correlations with some metabolic pathways, the contextualized embedding dimensions capture stronger correlation, signified by the darker blue and red colors in the heatmap.

To assess the statistical significance of the observed correlations, we applied a permutation test with 1,000 permutations. This test generates a distribution of correlations under the null hypothesis that the embeddings and the pathways are independent. By comparing the observed correlations to this null distribution, we filtered out correlations that were not statistically

significant. We then compare the strengths of the remaining statistically significant correlations found for our contextualized embeddings to those found for the GloVe embeddings, by contrasting the distribution of the filtered correlation magnitudes from both embeddings in Figure 7, which visually shows that the normalized histograms of the contextualized embedding dimensions are shifted to the right compared to that of the GloVe embedding dimensions.

## Code

First, it is a bit troubling that the code was not made directly available, and apparently was not assessed in the first round of reviews.

We apologize for the oversight that delayed the Dryad repository from becoming publicly accessible. That issue is now resolved, and the data and code for the current submission of our paper are now available at https://datadryad.org/stash/dataset/doi:10.5061/dryad.tb2rbp08p.

Second, the code provided by the authors lacks adequate documentation, making it difficult for other researchers to reproduce the work. While the README does describe each file in detail, there is no documentation on how one would utilize the files and code to reproduce the work (e.g., train the model or evaluate the model) or apply the authors' model to novel datasets. Moreover, the code itself generally lacks documentation (e.g., function-level documentation on the purpose of the function, input parameters, and output). Comprehensive documentation is essential for ensuring that the research can be replicated and that the developed tools can be effectively applied to new research goals. The lack of clear instructions and comments in the codebase detracts from the overall impact of this work.

We have updated the code to match the experiments described in the current submission, added significant additional documentation of code functionality, as well as clarified the steps required to reproduce our results and to apply our method to novel datasets.

## Computation

The authors do not state the amount of computation required for training and inference. This information is crucial in evaluating the feasbility of the authors' approach, especially when scaled to larger datasts.

Our original submission contained this information in the supporting information, but we unfortunately neglected to mention this in the main body of the paper. In our revision, we have moved the table for computation time (Table 2) to the main body of the text. Specifically, we document the time needed for various experiments (using a single Nvidia A40 GPU) as follows:

- Generator pretraining for 240 epochs: 19.3 hours
- Discriminator pretraining for 120 epochs: 4.13 hours
- IBD fine-tuning with 5 runs: 12.20 hours
- Fruit fine-tuning with 5 runs: 13.98 hours

- Vegetable fine-tuning with 5 runs: 10.74 hours

# Minor Comments

Introduction: The repeated use of "bacteria" in the Abstract and Introduction is misleading, as the dataset includes both bacteria and archaea (at least, there is not mention of filtering out archaeal ASVs). The authors should ensure that the archaeal component is appropriately acknowledged.

Thank you for pointing out this oversight. The precise microbial breakdown of our vocabulary entries are as follows: 26,546 bacteria, 113 eukaryota, 42 complete unknowns, and 25 archaea. We have edited our manuscript to use the word "microbe" in place of "bacteria".

Line 44: The statement "enables contextualized interpretation of individual bacterial species" is misleading since ASVs are not equivalent to species taxonomic units, as defined by standardized taxonomies such as GreenGenes, SILVA, or GTDB. The same goes for any further mention of ASVs as "species".

We have corrected our usage of the word "species" to describe ASVs.

Line 77: The analogy comparing microbiome data to textual sentences is flawed since microbiome samples do not have an inherent ordering like syntax in sentences. This analogy should be revised or omitted.

We agree that there are differences between the microbiome data and textual data, notably the lack of sequential nature in the microbiome data. However, we believe the resemblance between the two domains, namely the contextual sensitivity of the taxa, is important toward illuminating the suitability of the transformer model as well as the mask-based pre-training strategy for microbiome data. These two ideas both originated from the NLP domain, hence we would like to retain this analogy, but with an added comment noting the distinction between the two domains.

Line 174: Typographical error, ".." should be corrected to ".".

We have corrected this mistake.

Line 176: Clarification is needed on which samples from the AGP dataset were used to train the GloVe model. If samples used for training were also included in the test splits, data leakage has occurred. According to Tataru and David, 2020: "By applying the GloVe algorithm to 18,480 gut microbiome samples from the American Gut Project…". Thus, it appears that GloVe was trained on all 18480 AGP samples used in this work.

We have clarified that all methods in our study were allowed equal access to the full AGP dataset for their unsupervised training stages.

Line 235: The term "each taxa" should be corrected to "each taxon."

We have fixed this mistake.

Line 273: Clarify whether fine-tuning was performed only with the AGP dataset or on each dataset in the study.

We have clarified that we only finetune the models on splits of the AGP data.

Line 289: Reiterate that ASVs are not equivalent to microbial species as defined by established taxonomies.

We have fixed this mistake.

Line 308: State whether all samples are from different individuals or if there are repeated observations. If there are repeated observations, clarify whether individuals were split between training and validation datasets.

We have edited the dataset descriptions to mention that there are repeated observations for individuals in all three datasets, and that we do not split datasets by individual for the AGP dataset.

Line 329: Confirm the presence of repeated observations in the Halfvarson dataset and detail the distribution of these repeated observations and how non-independence was handled during model fine-tuning.

We have clarified that we do not finetune on either Halfvarson or HMP2, and so there is no train-test split for which to block out datapoints by individual.

Line 432: Quantify the correspondence between ASV distances in embedding and taxonomic space to prevent incorrect conclusions from t-SNE or UMAP projections.

Our prior manuscript included a clustering analysis in Figure 5 part d), which took place in the full embedding space with no dimension reduction, and therefore does not rely on t-SNE. It shows that ASVs more strongly cluster by phylum when represented by the contextualized embedding, as compared to the GloVe embedding.

Line 479: It is unclear if the embeddings significantly differ; neither Figure 6 (heatmap) nor Figure 7 (histogram) convincingly demonstrate this.

We have added two non-parametric two-sample statistical tests (Kolmogorov–Smirnov and Epps–Singleton), which both decisively reject the null hypothesis that the two distributions of correlations are identical.

Line 488: “each taxa” should be corrected to “each taxon” or “each ASV” for accuracy.

We have corrected this mistake in the text.

Line 496: The statement’s validity is questionable due to potential artifacts from t-SNE and UMAP. Consider a more quantitative approach, such as clustering embeddings and assessing the distribution of attribution values among clusters.

We have qualified our statement in the manuscript to reflect that it is subject to the capabilities and limitations of the t-SNE method. We also added a brief discussion of those capabilities and limitations (capturing local pairwise distances / neighborhood structure, but fails to accurately reflect longer distance relationships and global structure) when first introducing t-SNE.

Tables 4 & 5: Provide ASV identifiers and/or 16S sequences so that researchers can utilize this information for future work.

We have added tables 7 and 8 to the Supporting Information with the 16S sequences associated with the ASVs most associated with health and disease, respectively.

Figure 6: Specify the measure of correlation used.

We have clarified that we used Spearman’s correlation.

Bibliography

Bradley, A.P. (1997). The use of the area under the ROC curve in the evaluation of machine learning algorithms. *Pattern Recognit., 30*, 1145-1159.
